# A PUF-Based Secure and Lightweight Authentication Protocol for Medical IoT Environments

**DOI:** 10.3390/s26103223

**Published:** 2026-05-19

**Authors:** Hyeongeun Lim, Yohan Park, Youngho Park

**Affiliations:** 1 School of Electronic and Electrical Engineering, Kyungpook National University, Daegu 41566, Republic of Korea; lim886@knu.ac.kr; 2 School of Computer Engineering, Keimyung University, Daegu 42601, Republic of Korea

**Keywords:** lightweight authentication, Medical Internet of Things (MIoT), Physical Unclonable Function

## Abstract

The development of sensor and communication technology has enabled the Internet of Things in healthcare. In Medical Internet of Things (MIoT) environments, sensors support real-time patient monitoring, remote diagnosis, and early disease detection. However, communication between users and sensors over public channels is vulnerable to various security attacks, making secure and lightweight authentication with session key establishment essential for protecting medical data. Recently, a lightweight and anonymous authentication protocol for MIoT environments was proposed using Physical Unclonable Functions (PUFs); however, we show that their protocol is vulnerable to eavesdropping, stolen verifier, and ephemeral secret leakage attacks, and fails to guarantee untraceability. To address these weaknesses, we propose a secure and lightweight PUF-based authentication protocol for MIoT environments. The security of our protocol is formally verified using Burrows–Abadi–Needham logic, the Real-or-Random model, and the Scyther tool. Furthermore, the practical validation of the proposed protocol is conducted on a hardware platform along with an evaluation of energy consumption based on the MIRACL cryptographic library. Performance comparisons demonstrate that our protocol achieves enhanced security properties with minimal computational overhead and communication costs. Ultimately, this research provides a secure and robust architectural option for healthcare applications aiming to preserve patient privacy in resource-constrained MIoT.

## 1. Introduction

With the rapid advancement of communication and sensor technology, the Internet of Things (IoT) has been applied across diverse domains, particularly in healthcare, leading to the emergence of the Medical Internet of Things (MIoT) [1]. The MIoT network architecture integrates wearable sensors, smart devices, and communication technology to provide medical services [2]. In this environments, sensors are utilized for patient monitoring, remote diagnosis, and disease management [3]. The medical data collected by these sensors are delivered to medical staff through gateways or servers. Then, the medical staff receive and analyze medical data via mobile devices to make clinical decisions [4]. Thus, MIoT has become a crucial part of future healthcare systems by improving the quality of medical services, reducing response times, and supporting personalized treatment.

Despite these advantages, MIoT environments are susceptible to significant security and privacy concerns. Communication in MIoT environments is conducted over public channels; thus, sensitive medical data can be exposed to various security threats [5]. In addition, as sensors are low specification devices with restricted battery capacity, storage space, and computational power, applying heavy cryptographic operations in MIoT environments is challenging [6]. An adversary can eavesdrop on communications, intercept transmitted messages, impersonate legitimate participants, or carry out other malicious activities, potentially resulting in identity theft, privacy leakage, and even medical fraud [7]. Therefore, lightweight authentication and secure session key establishment are required for security and efficiency in MIoT environments.

In 2025, Lee et al. [8] proposed a lightweight authentication protocol for MIoT using a Physical Unclonable Function (PUF), hash functions, and exclusive-OR (XOR) operations. Their protocol aimed to provide resistance against attacks and user untraceability while maintaining low computational overhead. However, our cryptanalysis demonstrates that their protocol is susceptible to eavesdropping, stolen verifier, and ephemeral secret leakage (ESL) attacks, while failing to provide untraceability. Therefore, we suggest an authentication protocol that ensures user untraceability, resists stolen verifier and ESL attacks, and provides mutual authentication. The proposed protocol also provides lightweight performance suitable for low specification medical devices. Accordingly, this study aims to determine whether a lightweight authentication and key agreement protocol can achieve both security and low computational and communication overhead for resource-constrained medical devices.

### 1.1. Contributions

- We cryptanalyze that Lee et al.’s protocol [8] is susceptible to eavesdropping attacks, ESL attacks, and stolen verifier attacks, and fails to guarantee untraceability.
- We propose a secure lightweight authentication protocol based on the PUF and hash/XOR operations for MIoT environments.
- We formally verify the security of the proposed protocol using “the Real-or-Random (ROR) model” [9,10], “Burrows–Abadi–Needham (BAN) logic” [11], and “the Scyther tool” [12].
- We evaluate our protocol’s performance based on the MIRACL cryptographic library [13] and compare it with existing related protocols regarding computational cost, communication overhead, and security properties.

### 1.2. Organization

This paper is organized into nine sections. Section 2 introduces the related work in MIoT environments. Next, Section 3 presents the threat model, PUF, and system model. Section 4 reviews Lee et al. [8]’s protocol, and Section 5 provides the cryptanalysis of the protocol and reveals its vulnerabilities. Then, Section 6 proposes an improved protocol that addresses security vulnerabilities, and Section 7 evaluates the security analysis using BAN logic, the ROR model, and the Scyther tool. Section 8 presents cost and security comparison with existing protocols. Lastly, Section 9 presents the concluding remarks.

## 2. Related Works

In 2016, Dimitrov [14] introduced the MIoT framework in which sensors, wearable devices, and medical equipment are interconnected with cloud-based electronic medical records. In such environments, sensitive data (e.g., electrocardiogram signals, blood pressure measurements, and medication histories) are transmitted over public channels, requiring robust authentication and key exchange protocols [15]. In recent decades, various authentication protocols have been suggested for MIoT environments on Wireless Sensor Networks (WSN). Table 1 compares the authentication protocols discussed in this section with the proposed scheme in terms of their design approaches and key security properties.

In 2021, Subramani et al. [16] suggested an authentication protocol using a fuzzy extractor and PUFs, but Subramani et al.’s protocol incurs high computational overhead. Then, in 2022, Shao et al. [17] introduced another authentication protocol in MIoT using PUFs and a fuzzy extractor. However, this protocol suffers from high communication costs. In addition, Alladi et al. [18] suggested a two-way authentication protocol for healthcare IoT network. Alladi et al.’s protocol implements a two-stage authentication step between the sensor and server, and between the server and patient. In 2024, Modarres et al. [19] performed a cryptanalysis demonstrating that Alladi et al.’s protocol [18] is susceptible to eavesdropping, man-in-the-middle (MitM), and impersonation attacks. Hence, Modarres et al. proposed a PUF-based authenticated key exchange protocol for cloud based MIoT environments. Modarres et al.’s protocol applies a cloud-based MIoT network model with a three-layer architecture consisting of patient body sensors, a wireless node, and a cloud server to support secure communication. Recently, several studies have pointed out that MIoT data processing is not limited to a cloud-centric model [27]. In particular, edge computing and the computing continuum have been regarded as viable architectural options for supporting low-latency, real-time, and flexible healthcare services [28].

In 2020, Chen et al. [20] proposed a three-party mutual authentication and key exchange protocol for IoT environments based on WSN. They stated that their protocol resists both stolen verifier and insider attacks and ensures anonymity. However, in 2022, Hu et al. [21] proved that Chen et al.’s protocol is susceptible to impersonation attacks and fails to provide anonymity and unlinkability. Thus, they suggested a new two-factor authentication scheme. Their scheme employs a smart card to enable secure communication between the gateway and sensor node. Hu et al. claimed that their proposed protocol resists sensor node capture attacks. In 2024, Huang [22] introduced an authentication protocol using elliptic curve cryptography (ECC). They proposed an ECC based three factor authentication method based on passwords, smart cards and biometrics. However, ECC requires significantly more computational power than hash and XOR operations, making it less suitable for resource-constrained MIoT devices.

To reduce computational overhead, many lightweight protocols employ hash functions and XOR operations. Although these operations may be susceptible to mathematical analysis attacks, several studies have confirmed their security. Turkanovic et al. [23] proposed a hash and XOR-based authentication protocol for WSNs. Benfilali et al. [24] and Hussein et al. [25] presented lightweight hash and XOR-based authentication protocols for IoT. Safkhani et al. [26] further showed that lightweight hash based protocols combined with PUF mechanisms can resist eavesdropping, replay, and impersonation attacks. In 2025, Lee et al. [8] similarly adopted a lightweight authentication protocol for MIoT and showed that sensor node impersonation in Hu et al.’s protocol enables unauthorized key exchange. However, our cryptanalysis reveals that Lee et al.’s protocol fails to provide untraceability and is susceptible to eavesdropping, stolen verifier, and ESL attacks. Therefore, we suggest a robust and lightweight authentication protocol for the MIoT environments.

## 3. Preliminaries

This section presents the system model, PUF, the adversary model for the proposed MIoT environment, and the notation used in Lee et al. [8]’s and our protocol.

### 3.1. System Model

**Figure 1:**
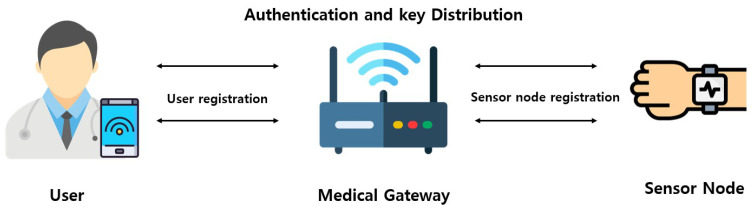
System model for the MIoT environments.

In this paper, we consider a three-entity MIoT system model comprising the user ($U_{i}$), the medical gateway (GWN), and the sensor node ($SN_{j}$). This system model was adopted to analyze the security and efficiency of the proposed protocol. Figure 1 presents the system model, and the details of each entity are described below.

- *User* ($U_{i}$): $U_{i}$ is a legal entity authorized to access patient medical information. $U_{i}$ register with the system through the gateway. $U_{i}$ can access patient medical data via mobile devices.
- *Gateway* (GWN): GWN is a trusted entity that mediates communication, authentication, and key distribution for all users and sensors. GWN possesses resources capable of performing complex operations.
- *Sensor node* ($SN_{j}$): $SN_{j}$ is registered through the gateway, and is a low-power device positioned on the body surface or internally in the patient. $SN_{j}$ collects medical information and transmits it to users via the gateway.

### 3.2. Physical Unclonable Function

In traditional systems, security keys are preserved in the software-defined memory and accessed during authentication or encryption. [29]. Adversaries may compromise stored secret keys using physical and logical attack vectors, including side-channel attacks. To mitigate these vulnerabilities, a PUF was introduced. The PUF exploits the intrinsic electrical randomness in semiconductor chip fabrication [30]. Given input, the PUF produces a chip-specific output. This relationship can be expressed as $R_{n}\leftarrow PUF\left(C_{n}\right)$, where $C_{n}$ denotes the *n*th challenge (input) and $R_{n}$ denotes the corresponding response (output). From a cryptographic perspective, a PUF must satisfy the following requirements [31].

- First, a PUF must reproduce nearly identical responses to the same challenge within an acceptable noise margin.
- Second, a PUF must generate sufficiently distinct responses across chips to ensure device-level uniqueness.
- Third, modeling and predicting responses from a subset of known challenge-response pairs (CRPs) must be computationally infeasible.

In the proposed protocol, the PUF generates authentication-related parameters in the registration phase and stores them in the server or gateway. In the authentication phase, the corresponding response value is applied to verify the message integrity and calculate the session key. When PUF stability is limited, environmental conditions (e.g., temperature or voltage fluctuations) may compromise response reproducibility. To address this issue, recent PUF designs provide stable responses across wide operating conditions [32,33]. The proposed protocol assumes the underlying PUF operates under stable conditions, ensuring consistent responses during authentication phases. If environmental noise causes an incorrect response, the ongoing authentication phase is terminated and a new phase is initiated. This design prevents an attacker from exploiting invalid responses to impersonate an authorized entity, thereby preserving the robustness of mutual authentication and key agreement in MIoT environments.

### 3.3. Adversary Model

We employ an widely-used “Dolev-Yao (DY) model [34]” and “Canetti-Krawczyk (CK) model [35]” in which the adversary *A* has the following capabilities.

- *A* has complete control over a public channel and can intercept, modify, delete, store, forge, or replay messages transmitted through a this channel [36]. *A* can use the collected messages to perform security attacks, including eavesdropping, MitM, and denial-of-service (DoS) attacks [37].
- *A* can compromise a user’s mobile device and extract certain security parameters stored within it using power analysis attacks [38]. Furthermore, *A* can acquire ephemeral secret values after session termination or obtain the long-term secret key of the server, enabling attacks (e.g, ESL) [39].
- *A* can perform polynomial-time computations to derive the user’s identity or password. However, the user’s identity, password, and device-stored data cannot be obtained simultaneously.

### 3.4. Protocol Notation

Table 2 list the notation employed in Lee et al. [8]’s and our protocol.

## 4. Review of Lee et al.’s  Protocol

In this section, we review the Lee et al. [8]’s protocol, which comprises the following phases: $U_{i}$ and $SN_{j}$ registrations, login and authentication, and password update. The details of Lee et al. [8]’s protocol are discussed follow.

### 4.1. User Registration Phase

**Figure 2:**
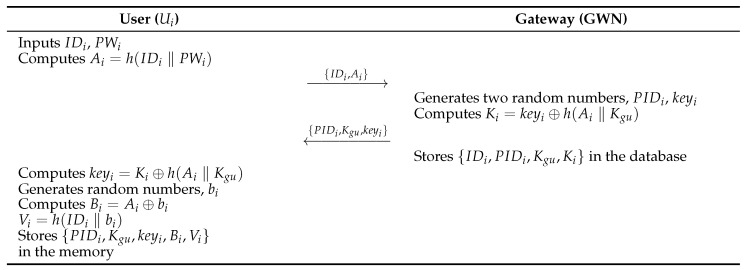
User registration phase of Lee et al. [8]’s protocol.

In this phase, $U_{i}$ register to GWN through a secure channel. The registration phase consists of the following steps. Figure 2 illustrates this phase.

**Step 1:** 
User $U_{i}$ enters $ID_{i}$ and $PW_{i}$ into the mobile device and calculates $A_{i}=h(ID_{i}||PW_{i})$.
**Step 2:** 
GWN generates two random values $PID_{i}$ and $key_{i}$ after receiving a message from $U_{i}$ and calculates $K_{i}=key_{i}⊕h(A_{i}\left|\right|K_{gu})$. GWN stores $\left\{ID_{i},PID_{i},K_{gu},K_{i}\right\}$ and sends the message $\left\{PID_{i},K_{gu},K_{i}\right\}$ to $U_{i}$.
**Step 3:** 
$U_{i}$ receives $\left\{PID_{i},K_{gu},K_{i}\right\}$ from GWN, and selects a random number $b_{i}$. Next, GWN calculates $key_{i}=K_{i}⊕h\left(A_{i}∥K_{gu}\right)$, $B_{i}=A_{i}⊕b_{i}$ and $V_{i}=h\left(ID_{i}∥b_{i}\right)$. Then $U_{i}$ stores $\left\{PID_{i},K_{gu},B_{i},V_{i}\right\}$ in the user’s mobile device memory.

### 4.2. Sensor Registration Phase

**Figure 3:**
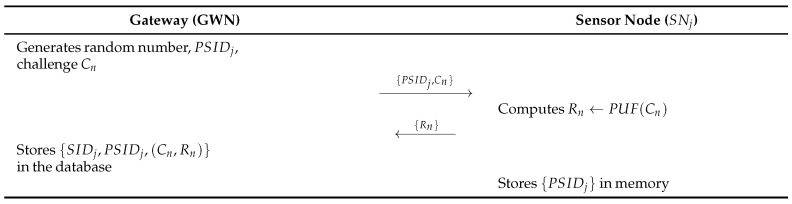
Sensor registration phase of Lee et al. [8]’s protocol.

In this phase, $SN_{j}$ must be registered with GWN through a secure channel. The sensor node registration phase consists of the following steps. Figure 3 illustrates this phase.

**Step 1:** 
GWN selects a random number $PSID_{i}$ and a challenge $C_{n}$ and send them to $SN_{j}$.
**Step 2:** 
$SN_{j}$ computes $R_{n}\leftarrow PUF\left(C_{n}\right)$ and sends $R_{n}$ to GWN.
**Step 3:** 
$SN_{j}$ stores $PSID_{i}$ in the memory.

### 4.3. Login and Authentication Phase

**Figure 4:**
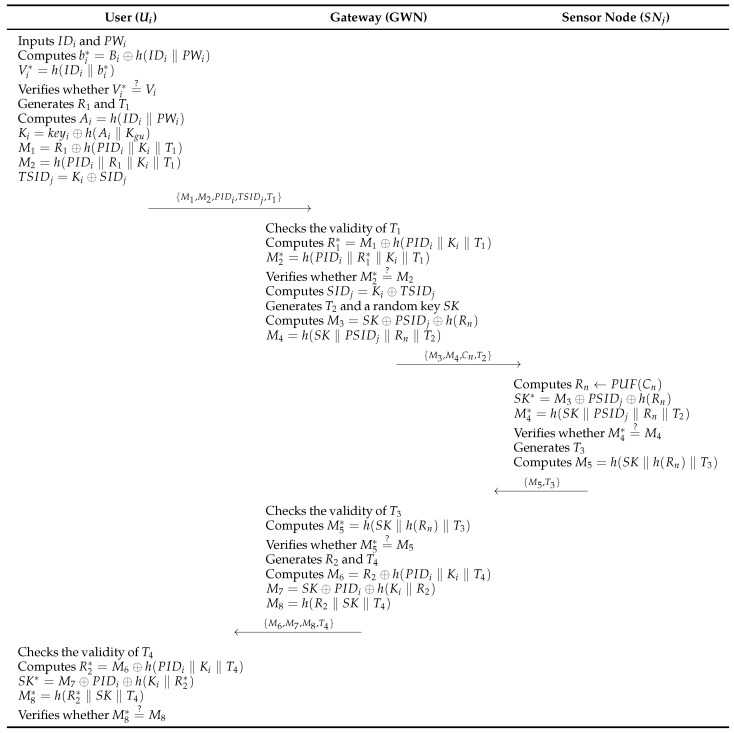
Login and the authentication phase of Lee et al. [8]’s protocol.

In the login and authentication phase, $U_{i}$, GWN and $SN_{j}$ perform authentication and generate a session key SK. The login and authentication phase consists of the following steps. Figure 4 illustrates this phase.

**Step 1:** 
$U_{i}$ inputs $ID_{i}$ and $PW_{i}$ into the mobile device, calculates $b_{i}^{*}=B_{i}⊕h\left(ID_{i}\|PW_{i}\right)$ and $V_{i}^{*}=h\left(ID_{i}\|b_{i}^{*}\right)$, verifies whether $V_{i}^{*}\overset{?}{=}V_{i}$. If the validity is confirmed, $U_{i}$ selects a random nonce $R_{1}$ and timestamp $T_{1}$, and computes $A_{i}=h\left(ID_{i}\|PW_{i}\right)$, $K_{i}=key_{i}⊕h\left(A_{i}\|K_{gu}\right)$, $M_{1}=R_{1}⊕h(PID_{i}\|K_{i}\left\|T_{1}\right)$, $M_{2}=h(PID_{i}\|R_{1}\|K_{i}\left\|T_{1}\right)$, and $TSID_{j}=K_{i}⊕SID_{j}$, and sends $\left\{M_{1},M_{2},PID_{i},TSID_{j},T_{1}\right\}$ to GWN.
**Step 2:** 
When GWN receives $\left\{M_{1},M_{2},PID_{i},TSID_{j},T_{1}\right\}$ from $U_{i}$, GWN validates the freshness of $T_{1}$, calculates $R_{1}^{*}=M_{1}⊕h(PID_{i}\|K_{i}\left\|T_{1}\right)$ and $M_{2}^{*}=h(PID_{i}\|R_{1}^{*}\|K_{i}\left\|T_{1}\right)$, and verifies whether $M_{2}^{*}\overset{?}{=}M_{2}$. Next, GWN recovers $SID_{j}=K_{i}⊕T.SID_{j}$, generates a random session key SK and timestamp $T_{2}$, computes $M_{3}=SK⊕PSID_{j}⊕h\left(R_{n}\right)$ and $M_{4}=h(SK\|PSID_{j}\|R_{n}\left\|T_{2}\right)$, and forwards $\left\{M_{3},M_{4},C_{n},T_{2}\right\}$ to $SN_{j}$.
**Step 3:** 
$SN_{j}$ receives $\left\{M_{3},M_{4},C_{n},T_{2}\right\}$ from GWN and checks the validity of $T_{2}$. $SN_{j}$ computes $R_{n}\leftarrow PUF\left(C_{n}\right)$, $SK^{*}=M_{3}⊕PSID_{j}⊕h\left(R_{n}\right)$, and $M_{4}^{*}=h(SK^{*}\|PSID_{j}\|R_{n}\left\|T_{2}\right)$, and verifies whether $M_{4}^{*}\overset{?}{=}M_{4}$. Afterward, $SN_{j}$ generates timestamp $T_{3}$, computes $M_{5}=h(SK\|h\left(R_{n}\right)\left\|T_{3}\right)$, and forwards $\left\{M_{5},T_{3}\right\}$ to GWN.
**Step 4:** 
When GWN receives $\left\{M_{5},T_{3}\right\}$ from $SN_{j}$, GWN checks the freshness of $T_{3}$ and computes $M_{5}^{*}=h(SK\|h\left(R_{n}\right)\left\|T_{3}\right)$, verifies whether $M_{5}^{*}\overset{?}{=}M_{5}$. Subsequently, GWN generates a random nonce $R_{2}$ and timestamp $T_{4}$, computes $M_{6}=R_{2}⊕h(PID_{i}\|K_{i}\left\|T_{4}\right)$, $M_{7}=SK⊕PID_{i}⊕h\left(K_{i}\|R_{2}\right)$, and $M_{8}=h(R_{2}\left\|SK\right\|T_{4})$, and sends $\left\{M_{6},M_{7},M_{8},T_{4}\right\}$ to $U_{i}$.
**Step 5:** 
$U_{i}$ receives $\left\{M_{6},M_{7},M_{8},T_{4}\right\}$ from GWN and checks the validity of $T_{4}$. Then, $U_{i}$ computes $R_{2}^{*}=M_{6}⊕h(PID_{i}\|K_{i}\left\|T_{4}\right)$, $SK^{*}=M_{7}⊕PID_{i}⊕h\left(K_{i}\|R_{2}\right)$, and $M_{8}^{*}=h(R_{2}\left\|SK\right\|T_{4})$, and verifies whether $M_{8}^{*}\overset{?}{=}M_{8}$. Afterward, $U_{i}$ and GWN update the user’s $PID_{i}$ as $PID_{i}^{n}=PID_{i}⊕R_{2}$, and GWN and $SN_{j}$ update the sensor node $PSID_{j}$ to $PSID_{j}^{n}=PSID_{j}⊕h\left(SK\right)$.

## 5. Cryptanalysis of Lee et al.’s Protocol

In this section, we analyze the security vulnerabilities of Lee et al. [8]’s protocol. Our cryptanalysis reveals that the protocol is vulnerable to eavesdropping attacks, stolen-verifier attacks, and ESL attacks, and fails to ensure user untraceability. The detailed analysis is presented below.

### 5.1. Eavesdropping Attack

With authentication messages and $SID_{j}$, *A* can calculate another legal user’s session key SK. Because Lee et al. [8]’s protocol relays a randomly generated session key using simple XOR operations, it fails to protect against eavesdropping. The steps are listed as below.

**Step 1:** 
*A* intercepts a message $\{M_{1},M_{2},PID_{i},TSID_{j},$$T_{1}\}$ by eavesdropping on legitimate user sessions. As *A* knows $SID_{j}$, it can compute $TSID_{j}=K_{i}^{A}⊕SID_{j}$, where $K_{i}$ is a shared secret value between $U_{i}$ and GWN.
**Step 2:** 
*A* intercepts a message $\left\{M_{6},M_{7},M_{8},T_{4}\right\}$ from a public channel. Then *A* compute $R_{2}^{A}=M_{6}⊕h(PID_{i}\left|\right|K_{i}\left|\right|T_{4})$, where $R_{2}^{A}$ represent a random number of gateway GWN. Finally, *A* calculates the session key $SK=M_{7}⊕PID_{i}⊕h(K_{i}\left|\right|R_{2})$.

Therefore, Lee et al. [8]’s protocol cannot prevent eavesdropping attacks.

### 5.2. User Untraceability

*A* can trace the user from the authentication messages. In particular, since the update of $PID_{i}$ involves only a simple XOR operation with $R_{2}$, *A* can correlate the pseudo identity across sessions. Thus, *A* remains capable of tracing $U_{i}$ by linking the old and new $PID_{i}$, even after the post-session update. The following steps detail the procedures for this attack.

**Step 1:** 
*A* eavesdrops on the public channel and obtains $\left\{M_{1},M_{2},PID_{i},TSID_{j},T_{1}\right\}$. Using $PID_{i}$, *A* traces the specific user.
**Step 2:** 
With the intercepted $TSID_{j}$, *A* computes $SID_{j}=TSID_{j}⊕K_{i}$, extracting the secret value $K_{i}$.
**Step 3:** 
*A* intercepts $\left\{M_{6},M_{7},M_{8},T_{4}\right\}$ from the public channel and computes $R_{2}^{A}=M_{6}⊕h(PID_{i}∥K_{i}∥T_{4})$. During the pseudo identity update, where $PID_{i}^{n}=PID_{i}⊕R_{2}$, *A* performs the same computation to trace the user.

Therefore, Lee et al. [8]’s protocol does not ensure user untraceability.

### 5.3. Stolen Verifier Attack

*A* computes the session key using stolen verification tables $\left\{ID_{i},PID_{i},K_{gu},K_{i}\right\}$ and $\left\{SID_{j},PSID_{j},\left(C_{n},R_{n}\right)\right\}$ and intercepted public channel messages. The steps for the attack process is as follows.

**Step 1:** 
*A* steals the GWN’s verification tables $\left\{ID_{i},PID_{i},K_{gu},K_{i}\right\}$ and $\left\{SID_{j},PSID_{j},\left(C_{n},R_{n}\right)\right\}$, obtaining the secret values $K_{i}$ and $PID_{i}$, which serve as the essential parameters for session key derivation.
**Step 2:** 
*A* captures $\left\{M_{6},M_{7},M_{8},T_{4}\right\}$ from the public channel and computes $R_{2}^{A}=M_{6}⊕h(PID_{i}∥K_{i}∥T_{4})$.
**Step 3:** 
Using the previously computed $R_{2}$ and stolen verification table, *A* calculates the session key as $SK=M_{7}⊕PID_{i}⊕h\left(K_{i}∥R_{2}\right)$.

### 5.4. Ephemeral Secret Leakage Attack

By intercepting the session-specific nonces $R_{1}$ and $R_{2}$, *A* becomes capable of deriving the session key. This attack scenario is executed through the following sequence.

**Step 1:** 
*A* intercepts $\left\{M_{1},M_{2},PID_{i},TSID_{j},T_{1}\right\}$ from a public channel by eavesdropping on the user’s session. Because $TSID_{j}=K_{i}^{A}⊕SID_{j}$ is known to *A*, the adversary can recover $K_{i}^{A}$ by computing $K_{i}^{A}=TSID_{j}⊕SID_{j}$.
**Step 2:** 
*A* intercepts $\left\{M_{6},M_{7},M_{8},T_{4}\right\}$ from a public channel. Using the obtained $K_{i}^{A}$ and $R_{2}$, the adversary can calculate $SK=M_{7}⊕PID_{i}⊕h\left(K_{i}^{A}∥R_{2}\right)$.

Therefore, Lee et al. [8]’s protocol fails to resist ESL Attack.

## 6. Proposed Protocol

We propose a PUF-based secure and lightweight authentication protocol for MIoT environments to overcome security vulnerabilities in the Lee et al. [8]’s protocol. Sensor nodes are low specification devices; hence, our protocol employs only hash functions, XOR operations, and PUFs to ensure low computational overhead. Our protocol comprises four phases: user registration, sensor node registration, authentication and key agreement, and password update.

### 6.1. User Registration Phase

**Figure 5:**
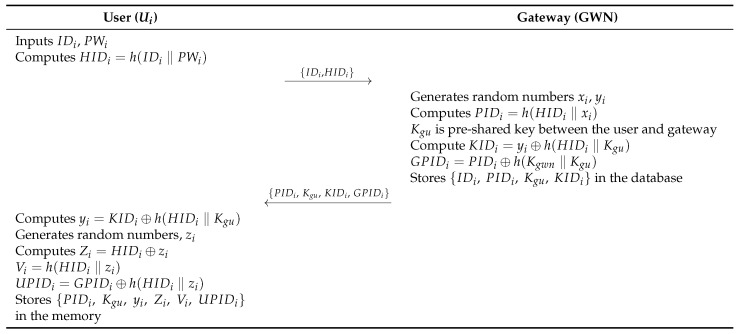
User registration phase of the proposed protocol.

In the user registration phase, $U_{i}$ sends a registration request to GWN. GWN generates secret parameters and sends them to the $U_{i}$’s mobile device through a secure channel. The user registration phase consists of the following steps. Figure 5 illustrates this phase.

**Step 1:** 
$U_{i}$ inputs $ID_{i}$ and $PW_{i}$, calculates $HID_{i}=h\left(ID_{i}\|PW_{i}\right)$, and sends $\left\{ID_{i},\;HID_{i}\right\}$ to GWN.
**Step 2:** 
GWN generates random nonces $x_{i}$ and $y_{i}$, where $K_{gu}$ denotes the pre-shared key between $U_{i}$ and GWN. GWN computes $PID_{i}=h\left(HID_{i}\|x_{i}\right)$, $KID_{i}=y_{i}⊕h\left(HID_{i}\|K_{gu}\right)$, and $GPID_{i}=PID_{i}⊕h\left(K_{gwn}\|K_{gu}\right)$ and stores $\left\{ID_{i},\;PID_{i},\;K_{gu},\;KID_{i}\right\}$ in the database, sending $\left\{PID_{i},\;K_{gu},\;KID_{i},\;GPID_{i}\right\}$ to $U_{i}$.
**Step 3:** 
$U_{i}$ calculates $y_{i}=KID_{i}⊕h\left(HID_{i}\|K_{gu}\right)$ and generates a random nonce $z_{i}$. Then, $U_{i}$ calculates $Z_{i}=HID_{i}⊕z_{i}$, $V_{i}=h\left(HID_{i}\|z_{i}\right)$, and $UPID_{i}=GPID_{i}⊕h\left(HID_{i}\|z_{i}\right)$. Finally, $U_{i}$ stores $\left\{PID_{i},\;K_{gu},\;y_{i},\;Z_{i},\;V_{i},\;UPID_{i}\right\}$ in the mobile device memory.

### 6.2. Sensor Registration Phase

**Figure 6:**
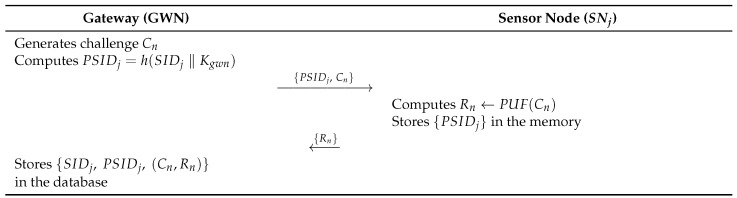
Sensor node registration phase of the proposed protocol.

In the sensor node registration phase, the GWN generates the parameters and registers $SN_{j}$ over a secure channel. The sensor registration phase consists of the following steps. Figure 6 illustrates this phase.

**Step 1:** 
GWN generates a challenge $C_{n}$. Then GWN computes $PSID_{j}=h\left(SID_{j}\|K_{gwn}\right)$. Then, GWN transmits $\left\{PSID_{j},C_{n}\right\}$ to $SN_{j}$.
**Step 2:** 
$SN_{j}$ computes $R_{n}\leftarrow PUF\left(C_{n}\right)$, and $SN_{j}$ stores $\left\{PSID_{j}\right\}$ in the memory. Subsequently, $SN_{j}$ transmits $R_{n}$ to GWN through a secure channel.
**Step 3:** 
GWN stores $\left\{SID_{j},\;PSID_{j},\;\left(C_{n},R_{n}\right)\right\}$ in the database.

### 6.3. Authentication and Key Agreement Phase

**Figure 7:**
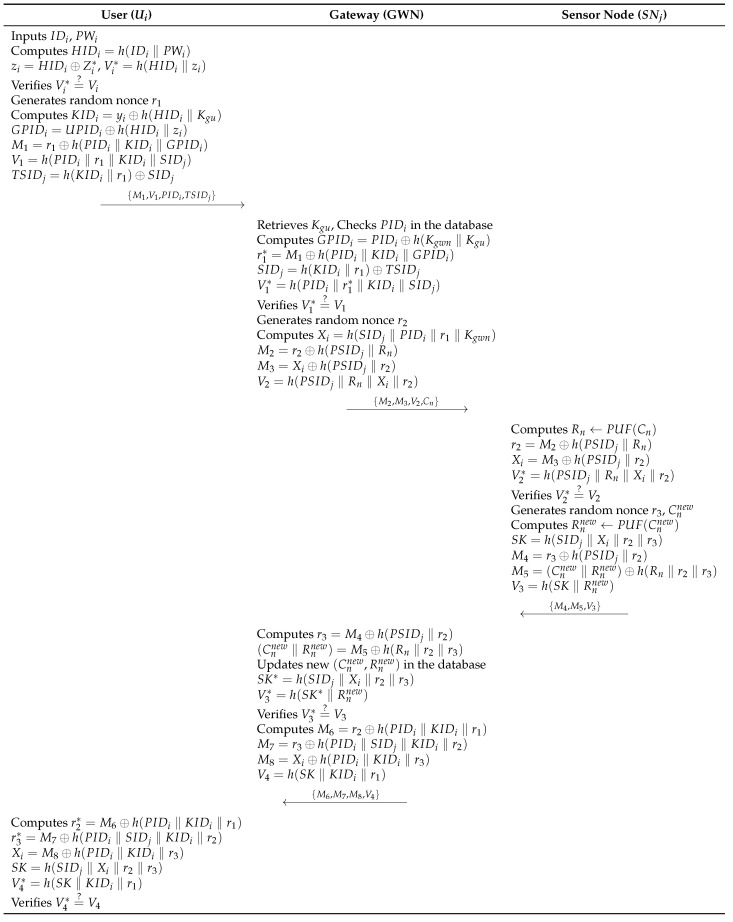
Authentication and key agreement phase of the proposed protocol.

This phase involves mutual authentication and session key agreement between $U_{i}$, GWN, and $SN_{j}$. The login and authentication phase consists of the following steps. Figure 7 illustrates this phase.

**Step 1:** 
$U_{i}$ inputs $ID_{i}$ and $PW_{i}$, and computes $HID_{i}=h\left(ID_{i}\|PW_{i}\right)$, $z_{i}=HID_{i}⊕Z_{i}^{*}$, and $V_{i}^{*}=h\left(HID_{i}\|z_{i}\right)$. Then, $U_{i}$ verifies the integrity by checking whether $V_{i}^{*}\overset{?}{=}V_{i}$. After verification, $U_{i}$ generates a random nonce $r_{1}$ and computes $KID_{i}=y_{i}⊕h\left(HID_{i}\|K_{gu}\right)$, $GPID_{i}=UPID_{i}⊕h\left(HID_{i}\|z_{i}\right)$, $M_{1}=r_{1}⊕h(PID_{i}\|KID_{i}\left\|GPID_{i}\right)$, $V_{1}=h(PID_{i}\|r_{1}\|KID_{i}\left\|SID_{j}\right)$, and $TSID_{j}=h\left(KID_{i}\|r_{1}\right)⊕SID_{j}$. Next, $U_{i}$ sends $\left\{M_{1},V_{1},PID_{i},TSID_{j}\right\}$ to GWN through a public channel.
**Step 2:** 
When GWN receives the message $\left\{M_{1},V_{1},PID_{i},TSID_{j}\right\}$, GWN retrieves $K_{gu}$ and checks $PID_{i}$ in the database. GWN computes $GPID_{i}=PID_{i}⊕h\left(K_{gwn}\|K_{gu}\right)$, $r_{1}^{*}=M_{1}⊕h(PID_{i}\|KID_{i}\left\|GPID_{i}\right)$, $SID_{j}=h\left(KID_{i}\|r_{1}\right)⊕TSID_{j}$ and $V_{1}^{*}=h(PID_{i}\|r_{1}^{*}\|KID_{i}\left\|SID_{j}\right)$, then verifies whether $V_{1}^{*}\overset{?}{=}V_{1}$. If the validity is confirmed, GWN generates a random nonce $r_{2}$, and computes $X_{i}=h(SID_{j}\|PID_{i}\|r_{1}\left\|K_{gwn}\right)$, $M_{2}=r_{2}⊕h\left(PSID_{j}\|R_{n}\right)$, $M_{3}=X_{i}⊕h\left(PSID_{j}\|r_{2}\right)$, and $V_{2}=h(PSID_{j}\|R_{n}\|X_{i}\left\|r_{2}\right)$. At last, GWN sends $\left\{M_{2},M_{3},V_{2},C_{n}\right\}$ to $SN_{j}$ over a public channel.
**Step 3:** 
After $SN_{j}$ receives $\left\{M_{2},M_{3},V_{2},C_{n}\right\}$, $SN_{j}$ calculates $R_{n}\leftarrow PUF\left(C_{n}\right)$ and recovers $r_{2}=M_{2}⊕h\left(PSID_{j}\|R_{n}\right)$ and $X_{i}=M_{3}⊕h\left(PSID_{j}\|r_{2}\right)$. $SN_{j}$ then calculates $V_{2}^{*}=h(PSID_{j}\|R_{n}\|X_{i}\left\|r_{2}\right)$ and verifies whether $V_{2}^{*}\overset{?}{=}V_{2}$. Then, $SN_{j}$ generates a random nonce $r_{3}$ and a new challenge $C_{n}^{new}$, and computes $R_{n}^{new}\leftarrow PUF\left(C_{n}^{new}\right)$, $SK=h(SID_{j}\|X_{i}\|r_{2}\|r_{3})$, $M_{4}=r_{3}⊕h\left(PSID_{j}\|r_{2}\right)$, $M_{5}=\left(C_{n}^{new}\|R_{n}^{new}\right)⊕h(R_{n}\|r_{2}\left\|r_{3}\right)$, and $V_{3}=h\left(SK\|R_{n}^{new}\right)$. $SN_{j}$ then sends $\left\{M_{4},M_{5},V_{3}\right\}$ to GWN over a public channel.
**Step 4:** 
When GWN receives messages $\left\{M_{4},M_{5},V_{3}\right\}$, GWN computes $r_{3}=M_{4}⊕h\left(PSID_{j}\|r_{2}\right)$ and recovers $\left(C_{n}^{new}\|R_{n}^{new}\right)=M_{5}⊕h(R_{n}\|r_{2}\left\|r_{3}\right)$. GWN then computes $SK^{*}=h(SID_{j}\|X_{i}\|r_{2}\left\|r_{3}\right)$ and $V_{3}^{*}=h\left(SK^{*}\|R_{n}^{new}\right)$, and checks whether $V_{3}^{*}\overset{?}{=}V_{3}$. Then, GWN updates $\left(C_{n}^{new},R_{n}^{new}\right)$ in the database and computes $M_{6}=r_{2}⊕h(PID_{i}\|KID_{i}\left\|r_{1}\right)$, $M_{7}=r_{3}⊕h(PID_{i}\|SID_{j}\|KID_{i}\left\|r_{2}\right)$, $M_{8}=X_{i}⊕h(PID_{i}\|KID_{i}\left\|r_{3}\right)$, and $V_{4}=h(SK\|KID_{i}\left\|r_{1}\right)$. GWN then sends $\left\{M_{6},M_{7},M_{8},V_{4}\right\}$ to $U_{i}$ over a public channel.
**Step 5:** 
After $U_{i}$ receiving the message $\left\{M_{6},M_{7},M_{8},V_{4}\right\}$, $U_{i}$ computes $r_{2}^{*}=M_{6}⊕h(PID_{i}\|KID_{i}\left\|r_{1}\right)$, $r_{3}^{*}=M_{7}⊕h(PID_{i}\|SID_{j}\|KID_{i}\left\|r_{2}\right)$, and $X_{i}=M_{8}⊕h(PID_{i}\|KID_{i}\left\|r_{3}\right)$. $U_{i}$ then computes $SK=h(SID_{j}\|X_{i}\|r_{2}\|r_{3})$ and $V_{4}^{*}=h(SK\|KID_{i}\left\|r_{1}\right)$, and verifies whether $V_{4}^{*}\overset{?}{=}V_{4}$.

After completing the authentication and key agreement phase, $U_{i}$, $SN_{j}$, and the GWN use the established session key SK for the current communication session. The session key is deleted when the current session terminates. If further communication is needed, the participating entities perform a new authentication and key agreement phase to establish a fresh session key.

### 6.4. The New Pseudo-Identity Update After Authentication

After the authentication and key agreement phase is completed, all entities update their new pseudo identities as below. This update is performed by utilizing the session key SK and random nonces $r_{1},r_{2},r_{3}$ generated during authentication process.

**Step 1:** 
$U_{i}$ and GWN calculate the new pseudo-identity of $U_{i}$ as $PID_{i}^{n}=h(PID_{i}\left\|SK\right\|r_{1}\left\|r_{2}\right)$, where the update utilizes SK along with $r_{1}$ and $r_{2}$.
**Step 2:** 
The user $U_{i}$ computes $UPID_{i}^{n}=UPID_{i}⊕PID_{i}⊕PID_{i}^{n}$, and replaces the previously stored values $\left\{PID_{i},UPID_{i}\right\}$ with the newly computed $\left\{PID_{i}^{n},UPID_{i}^{n}\right\}$.
**Step 3:** 
GWN and the sensor node $SN_{j}$ compute the new pseudo identity of $SN_{j}$ as $PSID_{j}^{n}=h(PSID_{j}\left\|SK\right\|r_{2}\left\|r_{3}\right)$. Both entities replace the old $PSID_{j}$ with $PSID_{j}^{n}$ in the memory, ensuring forward unlinkability for sessions.

### 6.5. Password Change Phase

**Figure 8:**
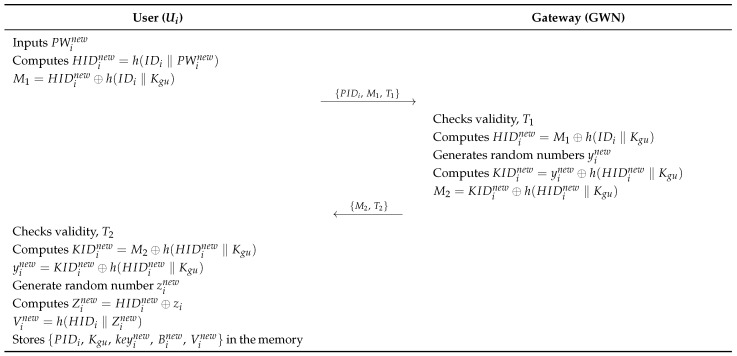
Password change phase of the proposed protocol.

In the password change phase, $U_{i}$ updates the new password by communicating with the GWN over a secure channel. Figure 8, illustrates the phases, and the detailed steps are described below.

**Step 1:** 
After entering a new password $PW_{i}^{new}$, $U_{i}$ derives $HID_{i}^{new}=h\left(ID_{i}\|PW_{i}^{new}\right)$ and $M_{1}=HID_{i}^{new}⊕h\left(ID_{i}\|K_{gu}\right)$, and transmits $\left\{PID_{i},\;M_{1},\;T_{1}\right\}$ to GWN.
**Step 2:** 
GWN verifies the validity of $T_{1}$ and calculates $HID_{i}^{new}=M_{1}⊕h\left(ID_{i}\|K_{gu}\right)$. Subsequently, GWN selects a random number $y_{i}^{new}$, calculates $KID_{i}^{new}=y_{i}^{new}⊕h\left(HID_{i}^{new}\|K_{gu}\right)$ and $M_{2}=KID_{i}^{new}⊕h\left(HID_{i}^{new}\|K_{gu}\right)$, and sends $\left\{M_{2},\;T_{2}\right\}$ to $U_{i}$.
**Step 3:** 
$U_{i}$ checks the validity of $T_{2}$ and calculates $KID_{i}^{new}=M_{2}⊕h\left(HID_{i}^{new}\|K_{gu}\right)$ and $y_{i}^{new}=KID_{i}^{new}⊕h\left(HID_{i}^{new}\|K_{gu}\right)$. $U_{i}$ also generates a random number $z_{i}^{new}$ and calculates $Z_{i}^{new}=HID_{i}^{new}⊕z_{i}$, $V_{i}^{new}=h\left(HID_{i}^{new}\|z_{i}^{new}\right)$, and $B_{i}^{new}=HID_{i}^{new}⊕z_{i}^{new}$. Finally, $U_{i}$ stores $\left\{PID_{i},\;K_{gu},\;key_{i}^{new},\;B_{i}^{new},\;V_{i}^{new}\right\}$ in the memory.

## 7. Security Analysis

### 7.1. Informal Security Analysis

The proposed protocol provides security against various attacks, including eavesdropping, stolen verifier, ESL, user device capture, privileged insider, and replay attacks, while ensuring untraceability. Moreover, protection against PUF modeling attacks is integrated into the protocol, thereby supporting secure mutual authentication. In addition, the informal security analysis considers impersonation, traceability, and secret leakage-related threats. In this context, side-channel leakage is regarded as a possible attack vector that may expose secret information and facilitate such attacks.

#### 7.1.1. Eavesdropping Attack

The adversary *A* attempts to compute the session key SK by eavesdropping on public messages $\left\{M_{1},V_{1},PID_{i},TSID_{j}\right\}$, $\left\{M_{2},M_{3},V_{2},C_{n}\right\}$, $\left\{M_{4},M_{5},V_{3}\right\}$, and $\left\{M_{6},M_{7},M_{8},V_{4}\right\}$ exchanged during the authentication phase. However, to compute $SK=h(SID_{j}\|X_{i}\|r_{2}\|r_{3})$, *A* must calculate the secret parameter $X_{i}=h(SID_{j}\|PID_{i}\|r_{1}\left\|K_{gwn}\right)$. But *A* cannot obtain the hash-masked random number $r_{1}$ or the gateway master key $K_{gwn}$. Therefore, our proposed protocol is resilient to insider attack.

#### 7.1.2. Privileged Insider Attack

A privileged insider adversary *A* intercepts registration request $\left\{ID_{i},PID_{i}\right\}$ and attempts to derive the session key $SK=h(SID_{j}\|X_{i}\|r_{2}\|r_{3})$ by employing the messages $\left\{M_{1},V_{1},PID_{i},TSID_{j}\right\}$, $\left\{M_{2},M_{3},V_{2},C_{n}\right\}$, $\left\{M_{4},M_{5},V_{3}\right\}$, and $\left\{M_{6},M_{7},M_{8},V_{4}\right\}$. To compute SK, *A* requires $KID_{i}$ to derive $r_{2}$, $r_{3}$, and $X_{i}$. By utilizing XOR operations and hash functions, these parameters are kept confidential, where $r_{2}=M_{2}⊕h\left(PSID_{j}\|R_{n}\right)$ and $KID_{i}=y_{i}⊕h\left(HID_{i}\|K_{gu}\right)$. The combination of $K_{gu}$, a registration-phase shared key between $U_{i}$ and GWN, and $y_{i}$, a random number, makes it computationally infeasible for A to extract $KID_{i}$. Hence, our protocol withstands privileged insider attacks.

#### 7.1.3. Untraceability and Anonymity

The following analysis confirms that the user’s identity is concealed and protected from tracing attacks throughout the login and authentication process. Instead of using the actual identity $ID_{i}$, GWN computes a pseudo-identity $PID_{i}=h\left(HID_{i}\|x_{i}\right)$. In Section 6.4, $PID_{i}$ is updated as $PID_{i}^{n}=h(PID_{i}\left\|SK\right\|r_{1}\left\|r_{2}\right)$ after each session to prevent identity tracing. Therefore, our protocol ensures untraceability and anonymity.

#### 7.1.4. Stolen Verifier Attack

Assume an *A* obtain the gateway’s verification tables $\left\{SID_{j},PSID_{j},\left(C_{n},R_{n}\right)\right\}$ and $\left\{ID_{i},P.ID_{i},K_{gu},K_{i}\right\}$ from the gateway and attempts to calculate the session key $SK=h(SID_{j}\|X_{i}\|r_{2}\|r_{3})$. However, to calculate SK, the attacker must know the gateway master key $K_{gwn}$ of the gateway and random numbers $r_{1},r_{2}$, and $r_{3}$. These values can only be obtained if *A* knows $KID_{i}=y_{i}⊕h\left(HID_{i}\|K_{gu}\right)$, where $HID_{i}=h\left(ID\|PW\right)$. Accordingly, our proposed protocol protocol is resilient to stolen verifier attacks.

#### 7.1.5. User Device Capture Attack

*A* captures a legal user’s mobile device and extracts the stored parameters $\left\{PID_{i},K_{gu},y_{i},Z_{i},V_{i},UPID_{i}\right\}$. Then, *A* attempts to compute the session key $SK=h(SID_{j}\|X_{i}\|r_{2}\|r_{3})$ using these stored parameters. However, the attacker must know $r_{2}$ and $r_{3}$, which are masked with $KID_{i}=y_{i}⊕h(h(ID||PW)\|K_{gu})$ and $PSID_{j}=h\left(SID_{j}\|K_{gwn}\right)$ to calculate the session key. Consequently, our protocol guarantees protection against user device capture attacks.

#### 7.1.6. Offline Password Guessing Attack

Suppose *A* extracts stored parameters $\left\{PID_{i},K_{gu},y_{i},Z_{i},V_{i},UPID_{i}\right\}$ from a legitimate user’s mobile device and attempts to guess the user’s password. As $Z_{i}=HID_{i}⊕z_{i}$, $V_{i}=h\left(HID_{i}\|z_{i}\right)$, and $UPID_{i}=GPID_{i}⊕h\left(HID_{i}\|z_{i}\right)$, where $HID_{i}=h\left(ID_{i}\|PW_{i}\right)$, the adversary cannot obtain the password without knowing the user’s identity $ID_{i}$. Furthermore, due to the property of the hash function, the reversal of these operations is infeasible. Hence, the proposed protocol ensures protection against offline password guessing attacks.

#### 7.1.7. Ephemeral Secret Leakage Attack

*A* obtains session nonces $r_{i}\left(i=1,\;2,\;3\right)$ and tries to derive the session key $SK=h(SID_{j}\|X_{i}\|r_{2}\|r_{3})$. However, computing SK without knowing $SID_{j}$ and $X_{i}$ is infeasible, as $X_{i}$ is masked with the pseudo identity $SID_{j}$ of the sensor node and the gateway master key $K_{gwn}$. Therefore, our protocol guarantees security against ESL attacks.

#### 7.1.8. Desynchronization Attack

An attacker blocks messages between the user, gateway, and sensor node during the login and authentication protocol. In the proposed protocol, sessions terminate when authentication values $V_{i}$ (i=1,2,3,4) do not match. In Section 6.4, new $PID_{i}$ and $PSID_{j}$ values are updated with the session key SK after session completion. Hence, our protocol resists desynchronization attacks.

#### 7.1.9. Impersonation Attack

*A* attempts to forge messages using the stored values derived from a user’s mobile device or sensor node, with messages observed from the sessions. For a mobile device impersonation attack, the adversary extracts $\left\{PID_{i},K_{gu},y_{i},Z_{i},V_{i},UPID_{i}\right\}$ and forge $\left\{M_{1},V_{1},PID_{i},TSID_{j}\right\}$. To do so, *A* must compute $y_{i}⊕h\left(HID_{i}\|K_{gu}\right)$, where $HID_{i}=h\left(ID\|PW\right)$, requiring ID and PW. For a sensor node impersonation attack, the adversary extracts $\left\{SPID_{j}\right\}$ and forges $\left\{M_{4},M_{5},V_{3}\right\}$. However, even if the adversary knows $C_{n}$, *A* cannot generate $R_{n}$ because the PUF produces a distinctive response for each sensor node. Thus, our protocol resists impersonation attacks.

#### 7.1.10. Perfect Forward Secrecy

Suppose *A* acquires the gateway’s master key $k_{gwn}$. *A* and attempts to calculate the session key $SK=h(SID_{j}\|X_{i}\|r_{2}\|r_{3})$. To calculate SK, *A* must obtain $X_{i}$, $SID_{j}$, and the random numbers $r_{2}$ and $r_{3}$. However, these values are masked by XOR operations and hash functions. Consequently, our protocol ensures perfect forward secrecy.

#### 7.1.11. PUF Modeling Attack

*A* collects a large data sets of challenge-response pairs from the PUF to predict them. In the proposed protocol, $\left(C_{n}^{\mathrm{new}}\|R_{n}^{\mathrm{new}}\right)=M_{5}⊕h(R_{n}\|r_{2}\left\|r_{3}\right)$ is masked with the random numbers $r_{2}$ and $r_{3}$ and hash functions, making it impossible to obtain the challenge pair. Additionally, new challenge–response pairs are updated at the gateway for each session to hinder inference. Moreover, the gateway transmits only $C_{n}$ to the sensor node. Therefore, our protocol is secure against PUF modeling attacks.

#### 7.1.12. Replay Attack

IF *A* intercepts a legitimate user’s messages $\left\{M_{1},V_{1},PID_{i},TSID_{j}\right\}$ and $\left\{M_{2},M_{3},V_{2},C_{n}\right\}$. Then, *A* can attempt to authenticate with the gateway and sensor in other sessions using these intercepted messages. Our proposed protocol verifies the freshness of $r_{1}$, $r_{2}$, and $r_{3}$ per session. Therefore, the proposed protocol resists replay attacks.

#### 7.1.13. Man-in-the-Middle Attack

*A* eavesdrops and modifies messages to attempt authentication. The verification values $V_{i}$ (i=1,2,3,4) detect attack, causing authentication failure and session termination. In addition, random numbers $r_{1}$, $r_{2}$ and $r_{3}$ ensure message freshness. Thus, our protocol resists MitM attacks.

#### 7.1.14. Denial of Service Attack

An attacker *A* continuously sends messages that appear valid to overload the gateway and sensor nodes. Our protocol applies verification values $V_{i}$ (i=1,2,3,4) with random numbers $r_{1},r_{2}$ and $r_{3}$ to check freshness. Thus, our protocol resists DoS attacks.

#### 7.1.15. Mutual Authentication

Throughout the proposed protocols, entities verify the values $V_{i}$ (i=1,2,3,4) each time they send messages. During the login and authentication phases, if any $V_{i}$ value is invalid, the session is terminated. If all $V_{i}$ values are valid, login and authentication are established. Consequently, our protocol ensures mutual authentication.

### 7.2. BAN Logic

BAN logic is a formal verification tool employed to analyze whether mutual authentication can be achieved in various authentication protocol [11]. We conduct a BAN logic-based proof to confirm that our protocol fulfills mutual authentication. The BAN logic notations are summarized in Table 3.

#### 7.2.1. Rules

The BAN logic analysis uses the following inference rules: Message Meaning Rule (MMR), Nonce Verification Rule (NVR), Jurisdiction Rule (JR), Belief Rule (BR), and Freshness Rule (FR), which are formally expressed below.

1. Message Meaning Rule (MMR):
  $$\frac{N_{i}|\equiv N_{i}\overset{K}{↔}N_{ii},\;N_{i}◃{\left\{S_{i}\right\}}_{K}}{N_{i}|\equiv N_{ii}|∼S_{i}}$$
2. Nonce Verification Rule (NVR):
  $$\frac{N_{i}|\equiv \#\left(S_{i}\right),\;N_{i}|\equiv N_{ii}|∼S_{i}}{N_{i}|\equiv N_{ii}|\equiv S_{i}}$$
3. Jurisdiction Rule (JR):
  $$\frac{N_{i}|\equiv N_{ii}\Rightarrow S_{i},\;N_{i}|\equiv N_{ii}|\equiv S_{i}}{N_{i}|\equiv S_{i}}$$
4. Belief Rule (BR):
  $$\frac{N_{i}|\equiv \left(S_{i},S_{ii}\right)}{N_{i}|\equiv S_{i}}$$
5. Freshness Rule (FR):
  $$\frac{N_{i}|\equiv \#\left(S_{i}\right)}{N_{i}|\equiv \#\left(S_{i},S_{ii}\right)}$$

#### 7.2.2. Goals

The BAN logic goals are presented as follows:

$$\begin{array}{cc} Goal_{1}: & \;U|\equiv U\overset{SK}{↔}GWN\\ Goal_{2}: & \;U|\equiv GWN|\equiv U\overset{SK}{↔}GWN\\ Goal_{3}: & \;GWN|\equiv U\overset{SK}{↔}GWN\\ Goal_{4}: & \;GWN|\equiv U|\equiv U\overset{SK}{↔}GWN\\ Goal_{5}: & \;SN|\equiv SN\overset{SK}{↔}GWN\\ Goal_{6}: & \;SN|\equiv GWN|\equiv SN\overset{SK}{↔}GWN\\ Goal_{7}: & \;GWN|\equiv SN\overset{SK}{↔}GWN\\ Goal_{8}: & \;GWN|\equiv SN|\equiv SN\overset{SK}{↔}GWN \end{array}$$

#### 7.2.3. Idealized Forms

To employ BAN logic, plaintext messages exchanged throughout authentication are converted into their abstract idealized representations, which are constructed as follows:

$$\begin{array}{cc} Msg_{1}:\; & U\to GWN:{\left\{r_{1},SID_{j}\right\}}_{KID_{i}}\\ Msg_{2}:\; & GWN\to SN:{\left\{r_{2},X_{i}\right\}}_{PSID_{j}}\\ Msg_{3}:\; & SN\to GWN:{\left\{r_{3}\right\}}_{PSID_{j}}\\ Msg_{4}:\; & GWN\to U:{\left\{r_{2},r_{3},X_{i}\right\}}_{KID_{i}} \end{array}$$

#### 7.2.4. Assumptions

Every participant assumes that random nonces and pseudo identities are fresh. After registration, each participant considers the shared secret keys established among the communicating entities to be valid. In addition, each participant believes that a legitimate entity can manage its own components and values. The proposed protocol is analyzed under the following BAN logic assumptions:

$$\begin{array}{cc} A_{1}: & GWN|\equiv \#(r_{1})\\ A_{2}: & SN|\equiv \#(r_{2})\\ A_{3}: & GWN|\equiv \#(r_{3})\\ A_{4}: & U|\equiv \#(X_{i})\\ A_{5}: & U|\equiv U\overset{KID_{i}}{↔}GWN\\ A_{6}: & GWN|\equiv U\overset{KID_{i}}{↔}GWN\\ A_{7}: & SN|\equiv SN\overset{PSID_{j}}{↔}GWN\\ A_{8}: & GWN|\equiv SN\overset{PSID_{j}}{↔}GWN\\ A_{9}: & U|\equiv GWN\Rightarrow (U\overset{SK}{↔}GWN)\\ A_{10}: & GWN|\equiv U\Rightarrow (U\overset{SK}{↔}GWN)\\ A_{11}: & SN|\equiv GWN\Rightarrow (SN\overset{SK}{↔}GWN)\\ A_{12}: & GWN|\equiv SN\Rightarrow (SN\overset{SK}{↔}GWN) \end{array}$$

#### 7.2.5. BAN Logic Proof

We establish the BAN logic assumptions below for our protocol in accordance with the foundational premises outlined above:

**Step 1:** $S_{1}$ is extracted from $Msg_{1}$.

$$GWN◃{\left\{r_{1},SID_{j}\right\}}_{KID_{i}}$$

**Step 2:** $S_{2}$ is derived by using the MMR to $S_{1}$ and $A_{6}$.

$$GWN|\equiv U|∼(r_{1},SID_{j})$$

**Step 3:** $S_{3}$ is derived by using the FR to $S_{2}$ and $A_{1}$.

$$GWN|\equiv \#(r_{1},SID_{j})$$

**Step 4:** $S_{4}$ is derived by using the NVR to $S_{3}$ and $S_{2}$.

$$GWN|\equiv U|\equiv (r_{1},SID_{j})$$

**Step 5:** $S_{5}$ is extracted from $Msg_{2}$.

$$SN◃{\left\{r_{2},X_{i}\right\}}_{PSID_{j}}$$

**Step 6:** $S_{6}$ is derived by using the MMR to $S_{5}$ and $A_{7}$.

$$SN|\equiv GWN|∼(r_{2},X_{i})$$

**Step 7:** $S_{7}$ is derived by using the FR to $S_{6}$ and $A_{2}$.

$$SN|\equiv \#(r_{2},X_{i})$$

**Step 8:** $S_{8}$ is derived by using the NVR to $S_{7}$ and $S_{6}$.

$$SN|\equiv GWN|\equiv (r_{2},X_{i})$$

**Step 9:** $S_{9}$ is extracted from $Msg_{3}$.

$$GWN◃{\left\{r_{3}\right\}}_{PSID_{j}}$$

**Step 10:** $S_{10}$ is derived by using the MMR to $S_{9}$ and $A_{8}$.

$$GWN|\equiv SN|∼(r_{3})$$

**Step 11:** $S_{11}$ is derived by using the FR to $S_{10}$ and $A_{3}$.

$$GWN|\equiv \#(r_{3})$$

**Step 12:** $S_{12}$ is derived by using the NVR to $S_{11}$ and $S_{10}$.

$$GWN|\equiv SN|\equiv (r_{3})$$

**Step 13:** $S_{13}$ is derived from $S_{12}$ and $S_{8}$. GWN and SN are capable of computing the $SK=h(SID_{j}\|X_{i}\|r_{2}\|r_{3})$.

$$SN|\equiv GWN|\equiv SN\overset{SK}{↔}GWN\;{(\mathrm{Goal}}_{6})$$

**Step 14:** $S_{14}$ is derived from $S_{12}$ and $S_{8}$. GWN and SN are capable of computing the $SK=h(SID_{j}\|X_{i}\|r_{2}\|r_{3})$.

$$GWN|\equiv SN|\equiv SN\overset{SK}{↔}GWN\;{(\mathrm{Goal}}_{8})$$

**Step 15:** $S_{15}$ is derived by using the JR to $S_{13}$ and $A_{12}$.

$$CS|\equiv SN\overset{SK}{↔}GWN\;{(\mathrm{Goal}}_{7})$$

**Step 16:** $S_{16}$ is derived by using the JR to $S_{14}$ and $A_{11}$.

$$SN|\equiv SN\overset{SK}{↔}GWN\;{(\mathrm{Goal}}_{5})$$

**Step 17:** $S_{17}$ is extracted from $Msg_{4}$.

$$U◃{\left\{r_{2},r_{3},X_{i}\right\}}_{KID_{i}}$$

**Step 18:** $S_{18}$ is derived by using the MMR to $S_{17}$ and $A_{5}$.

$$U|\equiv GWN|∼(r_{2},r_{3},X_{i})$$

**Step 19:** $S_{19}$ is derived by using the FR to $S_{18}$ and $A_{4}$.

$$GWN|\equiv \#(r_{2},r_{3},X_{i})$$

**Step 20:** $S_{20}$ is derived by using the NVR to $S_{19}$ and $S_{18}$.

$$U|\equiv GWN|\equiv (r_{2},r_{3},X_{i})$$

**Step 21:** $S_{21}$ is derived from $S_{9}$ and $S_{17}$. *U* and GWN are capable of computing the $SK=h(SID_{j}\|X_{i}\|r_{2}\|r_{3})$.

$$U|\equiv GWN|\Rightarrow \left(U\overset{SK}{↔}GWN\right)\;{(\mathrm{Goal}}_{2})$$

**Step 22:** $S_{22}$ is derived from $S_{9}$ and $S_{17}$. *U* and GWN are capable of computing the $SK=h(SID_{j}\|X_{i}\|r_{2}\|r_{3})$.

$$GWN|\equiv U|\Rightarrow \left(U\overset{SK}{↔}GWN\right)\;{(\mathrm{Goal}}_{4})$$

**Step 23:** $S_{23}$ is derived by using the JR to $S_{21}$ and $A_{9}$.

$$U|\equiv U\overset{SK}{↔}GWN\;{(\mathrm{Goal}}_{1})$$

**Step 24:** $S_{24}$ is derived by using the JR to $S_{22}$ and $A_{10}$.

$$GWN|\equiv U\overset{SK}{↔}GWN\;{(\mathrm{Goal}}_{3})$$

### 7.3. ROR Model

In this section, we conduct a formal security analysis of our protocol through the ROR model [9]. In the proposed protocol, $\Pi_{U}^{t_{1}}$, $\Pi_{G}^{t_{2}}$, and $\Pi_{S}^{t_{3}}$ denote the session entities of the user ($U_{i}$), gateway (GWN), and sensor node ($SN_{j}$), separately. The adversary A has the capability to eavesdrop on, intercept, delete, and insert messages [10]. Using these capabilities, A tries to calculate the session key through the Execute, CorruptSC, Reveal, Send, and Test queries. We describe precise descriptions of each query below.

- *Execute*$\left(\Pi_{U}^{t_{1}},\Pi_{G}^{t_{2}},\Pi_{S}^{t_{3}}\right)$: A eavesdrops on messages from a legitimate entity’s messages over a public channel. This query represents a passive attack.
- *CorruptMD*$\left(\Pi_{U_{1}}^{t}\right)$: This query simulates A who compromises the user’s mobile device to extract secret parameters stored in its memory, modeling physical device-capture attack.
- *Send*$\left(\Pi_{x}^{t},M\right)$: *A* transmits a message *M* to the instance $\Pi_{x}^{t}$ and captures the resulting response. This operation reflects the adversary’s capability to perform active communication attacks.
- *Test*$\left(\Pi_{x}^{t}\right)$: Through this oracle, A attempts to determine whether a challenge value corresponds to the actual session key SK or a random string. A random bit c∈{0,1} is flipped; if c=1, the genuine SK is provided to A, whereas if c=0, a random value of equivalent length is returned [40]. In cases of an invalid query, Null is issued. The protocol is considered secure if A can distinguish between these two cases with only a negligible advantage.

**Theorem** **1.** *A attempts to derive the session key SK in polynomial time. The notation ${\mathrm{Adv}}_{A}$ denotes the capability of A to obtain SK, with the following bound:*

(1) $$Adv_{A}\leq \frac{q_{h}^{2}}{\left|Hash\right|}+\frac{q_{p}^{2}}{\left|PUF\right|}+2\max\left\{C^{\prime }q_{send}^{s^{\prime }},\frac{q_{send}}{2^{l_{D}}}\right\}$$

*The query counts for hash, PUF, and send operations are denoted by $q_{h}$, $q_{p}$, and $q_{\mathrm{send}}$; the respective outputs are given by |Hash| and |PUF|. The constants $C^{\prime }$ and $s^{\prime }$ denote the parameters for “Zipf’s law [41]”.*

**Proof.**  To verify the security of the session key SK, we conduct a sequence of five games ${Game}_{k}$ (k=1,2,3,4,5). ${\mathrm{Succ}}_{k}$ denotes the event that A guess the correct bit in ${Game}_{k}$, and $\mathrm{Pr}[{\mathrm{Succ}}_{k}]$ (*k* = 0,i,ii,iii,iv) represents the probability of this event. We conduct the games in sequence.

$Game_{0}$: A attempts to guess a random bit. We formalize the advantage of A in this game as follows:

(2) $$Adv_{A}=\left|2\mathrm{Pr}\left[Succ_{0}\right]-1\right|$$

${Game}_{1}$: A attempts the *Execute* query to intercept on messages over the public channel. Since SK is masked by secret parameters, A cannot compute it through eavesdropping. Thus, the probability remains identical to ${Game}_{0}$ (2).

(3) $$\mathrm{Pr}\left[Succ_{i}\right]=\mathrm{Pr}\left[Succ_{0}\right]$$

${Game}_{2}$: A tries to calculate SK with the *Send* query. Even so, the session key is masked by secret parameters with a hash function, and obtaining the random nonce requires a separate *Hash* query. Thus, A’s advantage is limited by the birthday paradox of the hash function as follows [42]:

(4) $$|\mathrm{Pr}\left[Succ_{\mathrm{ii}}\right]-\mathrm{Pr}\left[Succ_{i}\right]|\leq \frac{q_{h}^{2}}{2|Hash|}$$

${Game}_{3}$: A tries to recover SK using the *Send* query. Like ${Game}_{\mathrm{ii}}$, ${Game}_{\mathrm{iii}}$ follows an identical birthday paradox structure for PUF queries, since both Hash and PUF produce outputs from fixed-size ranges susceptible to collision attacks. The advantage gap between $Game_{2}$ and $Game_{1}$ is given as follows:

(5) $$|\mathrm{Pr}\left[Succ_{\mathrm{iii}}\right]-\mathrm{Pr}\left[Succ_{\mathrm{ii}}\right]|\leq \frac{q_{p}^{2}}{2|PUF|}$$

$Game_{4}$: A uses the *CorruptMD* query to retrieve all private parameters from the mobile device and tries to calculate SK. SK computation requires recovering $PW_{i}$ to extract $z_{i}=Z_{i}⊕h(ID_{i}||PW_{i})$, which remains infeasible under “Zipf’s law [41]”. This yields Equation (7). The advantage gap between $Game_{3}$ and $Game_{4}$ is given as follows:

(6) $$|\mathrm{Pr}\left[Succ_{\mathrm{iv}}\right]-\mathrm{Pr}\left[Succ_{\mathrm{iii}}\right]|\leq \max\left\{C^{\prime }q_{send}^{s^{\prime }},\frac{q_{send}}{2^{l_{D}}}\right\}$$

(7) $$\mathrm{Pr}\left[Succ_{\mathrm{iv}}\right]=\frac{1}{2}$$

Equation (8) is derived from Equations (2), (3), and (7):

(8) $$\frac{1}{2}Adv_{A}=\left|\mathrm{Pr}\left[Succ_{0}\right]-\frac{1}{2}\right|=\left|\mathrm{Pr}\left[Succ_{i}\right]-\mathrm{Pr}\left[Succ_{\mathrm{iv}}\right]\right|$$

Using Equations (4)–(6) and the triangle inequality, we derive the following:

(9) $$\begin{array}{c} \frac{1}{2}{\mathrm{Adv}}_{A}=\left|\mathrm{Pr}\left[{\mathrm{Succ}}_{i}\right]-\mathrm{Pr}\left[{\mathrm{Succ}}_{\mathrm{iv}}\right]\right|\\ \leq \left|\mathrm{Pr}\left[{\mathrm{Succ}}_{i}\right]-\mathrm{Pr}\left[{\mathrm{Succ}}_{\mathrm{iii}}\right]\right|+\left|\mathrm{Pr}\left[{\mathrm{Succ}}_{\mathrm{iii}}\right]-\mathrm{Pr}\left[{\mathrm{Succ}}_{\mathrm{iv}}\right]\right|\\ \leq \left|\mathrm{Pr}\left[{\mathrm{Succ}}_{i}\right]-\mathrm{Pr}\left[{\mathrm{Succ}}_{\mathrm{ii}}\right]\right|+\left|\mathrm{Pr}\left[{\mathrm{Succ}}_{\mathrm{ii}}\right]-\mathrm{Pr}\left[{\mathrm{Succ}}_{\mathrm{iii}}\right]\right|+\left|\mathrm{Pr}\left[{\mathrm{Succ}}_{\mathrm{iii}}\right]-\mathrm{Pr}\left[{\mathrm{Succ}}_{\mathrm{iv}}\right]\right|\\ \frac{1}{2}{\mathrm{Adv}}_{A}\leq \frac{q_{h}^{2}}{2|Hash|}+\frac{q_{p}^{2}}{2|PUF|}+\max\left\{C^{\prime }q_{\mathrm{send}}^{s^{\prime }},\frac{q_{\mathrm{send}}}{2^{l_{D}}}\right\} \end{array}$$

Therefore, the final advantage of the adversary is summarized as:

(10) $$\;Adv_{A}\leq \frac{q_{h}^{2}}{\left|Hash\right|}+\frac{q_{p}^{2}}{\left|PUF\right|}+2\max\left\{C^{\prime }q_{send}^{s^{\prime }},\frac{q_{send}}{2^{l_{D}}}\right\}$$

□

### 7.4. Scyther Tool Simulation

In this section, we verify the security of the proposed protocol using the Scyther tool [12], a broadly used formal verification tool for security protocols. Scyther operates under the DY model, assuming that the communication channel is insecure and controlled by a malicious adversary. Rather than examining only a limited number of manually selected sessions, Scyther performs symbolic verification by systematically exploring possible protocol traces derived from the protocol roles and message flows. The protocol is modeled using the Security Protocol Description Language (SPDL), in which the roles of the user, gateway, and sensor node are formally defined. Scyther supports the verification of security properties such as secrecy and authentication through claim events, including secrecy, aliveness, and agreement-related claims. Table 4 describes the claim events employed to evaluate the security properties. These claims ensure that the protocol maintains secrecy and achieves mutual authentication.

**Figure 9:**
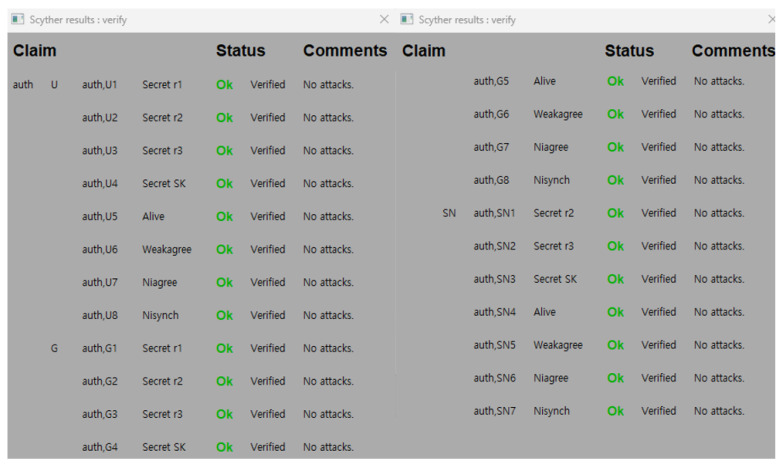
Scyther tool simulation result.

Scyther provides a graphical user interface to visualize the verification process. In Figure 9, the simulation results display “OK” in the status field and “No attacks” in the comments field for all specified claims. The Scyther verification results indicate that no attack trace was identified for the specified claims. Therefore, the Scyther analysis shows that the proposed protocol satisfies secrecy and mutual authentication in symbolic security verification.

## 8. Performance Comparison

In the performance comparison section, we evaluate the computational costs, communication costs, and security properties of the proposed protocol and existing protocols.

### 8.1. Computational Costs

In this section, we compare the computational costs during authentication of our proposed and related protocols. To evaluate Computational Cost, we conducted experiments on two hardware platforms: a Raspberry Pi 4 (ARM Cortex-A72 @ 1.5 GHz Quad-core, LPDDR4-3200 8 GB, Ubuntu 20.04 LTS, 64-bit) representing the resource-constrained User and Sensor Node, and an Intel Core i9-13900 Workstation (Intel Corporation, Santa Clara, CA, USA, assembled in China; 2.00–5.60 GHz, 24 cores, DDR5-4800 64 GB, Ubuntu 20.04 LTS, 64-bit) representing the Gateway. To quantify the computational cost of various cryptographic primitives on each platform, we utilized the MIRACL Crypto SDK [13], an open-source C/C++ based library compatible with diverse computing environments. The simulation code used in our experiments is publicly available [43]. The measured computational costs on the Raspberry Pi platform are as follows: ECC multiplication $T_{ECM}=2.133$ ms, fuzzy extractor generation $T_{Gen}=0.065$ ms, fuzzy extractor reproduction $T_{Rep}=0.060$ ms, hash function $T_{H}=0.005$ ms, and PUF challenge-response $T_{PUF}=0.025$ ms. For the Workstation (Gateway), the corresponding values are: $T_{ECM}=0.342$ ms, $T_{Gen}=0.013$ ms, $T_{Rep}=0.012$ ms, $T_{H}=0.001$ ms, and $T_{PUF}=0.005$ ms. The XOR operation computational time is excluded as it is trivial.

Table 5 presents our computation cost calculations result. In Lee et al. [8]’s protocol, the gateway generates a random session key and distributes it to each entity via XOR operations. In contrast, our protocol enhances security by computing the session key as $SK=h(SID_{j}\|X_{i}\|r_{2}\|r_{3})$ rather than simply relaying it. The proposed protocol incurs a higher computational cost than Lee et al.’s protocol [8]. However, the proposed protocol provides better security and performance than the other protocols.

### 8.2. Communication Costs

We analyze the communication costs of the proposed protocol and related protocols. Based on [16], the bit lengths for security parameters are defined as follows. The lengths of an ECC point, a hash function output, and a random nonce are set to 320 bits, 256 bits, and 256 bits, respectively. Furthermore, a 128-bit length is assigned to identities, passwords, and PUF challenge/response pairs, while the timestamp is allocated 32 bits. The proposed protocol exchanges four messages during authentication phase, with $\left\{M_{1},V_{1},PID_{i},TSID_{j}\right\}$, $\left\{M_{2},M_{3},V_{2},C_{n}\right\}$, $\left\{M_{4},M_{5},V_{3}\right\}$, and $\left\{M_{6},M_{7},M_{8},V_{4}\right\}$ costing 1024, 896, 768, and 1024 bits. Thus, the total communication cost is 1024+896+768+1024=3712 bits.

The proposed protocol introduces additional communication overhead because it includes the masking and transmission of parameters for secure session key establishment, whereas Lee et al.’s protocol adopts a simpler structure that directly relays the session key. Table 6 shows that the proposed protocol outperforms the protocols in [17,21,22].

Although the proposed protocol requires a larger communication overhead than Lee et al.’s protocol, the increase is not expected to affect the overall network load in practical MIoT environments. This is because authentication is performed only during session establishment, and representative standards such as IEEE 802.15.6 [44] and IEEE 802.15.4 [45] provide sufficient transmission capacity for low-power healthcare applications [46].

### 8.3. Energy Consumption

We compare the energy consumption of the proposed protocol against related works using the model defined in [47], where the total energy is $E_{total}=E_{C}+E_{M}$. Here, $E_{C}=C\times T_{k}$ is the computational energy with C=10.88 W and $T_{k}$ the total computation time, and $E_{M}=e_{bit}\times bit$ is the communication energy with $e_{bit}=0.00066$ mJ/bit. For the proposed protocol, the computation times are $9T_{H}=0.045$ ms (User), $15T_{H}=0.015$ ms (Gateway), and $2T_{PUF}+7T_{H}=0.085$ ms (Sensor Node), yielding $E_{C}=10.88\times 0.145=1.578$ mJ. With a communication cost of 3712 bits, $E_{M}=0.00066\times 3712=2.450$ mJ, giving $E_{total}=4.028$ mJ.

Table 7 summarizes the results. ECC-based protocols require higher energy costs: Hu et al. [21] consumes 100.406 mJ and Huang [22] consumes 175.764 mJ. Compared with existing PUF-based protocols, the proposed protocol reduces energy consumption by 44.2% and 38.8% compared to Subramani et al. [16] (7.208 mJ) and Shao et al. [17] (6.584 mJ), respectively. Although Lee et al. [8] achieves a lower energy of 2.805 mJ by simply relaying a randomly generated session key, the proposed protocol securely exchanges the parameters required for session key computation and derives the session key, requiring only a minimal overhead of 1.223 mJ with enhanced security, as demonstrated in Section 7.

### 8.4. Security Properties

A comparison of the security properties between the proposed protocol and other existing protocols [8,16,17,21,22] is summarized in Table 8. As shown in Table 8, the proposed protocol satisfies all the considered security properties, whereas the compared protocols fail to resist one or more attacks. In particular, several existing protocols are vulnerable to eavesdropping, stolen verifier, ESL, impersonation, MitM, and privileged insider attacks. Furthermore, only a limited number of existing schemes consider resistance to PUF modeling attacks, while the proposed protocol also provides protection against this threat. Therefore, the proposed protocol offers more comprehensive security than the existing protocols.

## 9. Conclusions

In this paper, we demonstrated that Lee et al.’s protocol is vulnerable to eavesdropping and stolen verifier attacks. We also verified that Lee et al.’s protocol does not guarantee untraceability. To overcome these security weaknesses, we suggested a lightweight and secure authentication protocol for MIoT environments based on PUF. Our proposed protocol resists various attacks, including eavesdropping attacks, privileged insider attacks, stolen verifier attacks, and ephemeral secret leakage attacks. Furthermore, it guarantees untraceability and provides secure mutual authentication and key agreement. Its security was formally verified using BAN logic, the ROR model, and the Scyther tool. By employing hash and XOR operations, the protocol achieves lightweight computation and communication efficiency. Furthermore, using PUF minimizes the computational cost on sensor nodes, making the protocol more secure and efficient for MIoT environments compared with existing protocols. These results demonstrate that a lightweight authentication and key agreement protocol can achieve both security and low computational and communication overhead for resource-constrained medical devices. In addition, the proposed protocol was validated on a Raspberry Pi-based hardware platform, including an evaluation of energy consumption. Despite these practical validations, this study has a limitation in that it assumes stable PUF operations without testing against extreme environmental noises. As future work, we plan to extend the protocol to support multi-gateway architectures within distributed edge and continuum computing environments. Ultimately, this research provides a secure and timely architectural option that is highly useful for engineers developing practical MIoT systems.

## Figures and Tables

**Table 1:** Feature comparison of authentication protocols for MIoT and WSN-based environments.

Year	Author	Technique	Contribution	Limitation
2022	Subramani et al. [16]	PUF, fuzzy extractor	Lightweight authentication for WBAN-based MIoT with three-factor verification	High computational cost
2022	Shao et al. [17]	PUF, fuzzy extractor	Anonymous authentication and session key agreement for MIoT	High communication cost
2021	Alladi et al. [18]	PUF, hash, XOR	Sensor–server and server–patient authentication for healthcare IoT networks	Eavesdropping, MitM, and impersonation
2024	Modarres et al. [19]	PUF, hash, XOR	Mutual authentication and key agreement for three-layer cloud-based MIoT	Cloud-centric; limited edge support
2020	Chen et al. [20]	Hash, XOR	Mutual authentication and key agreement for WSN-based IoT environments	Impersonation; no anonymity or unlinkability
2022	Hu et al. [21]	ECC, hash, XOR	Smart card-based secure communication between gateway and sensor node	Vulnerable to sensor node impersonation
2024	Huang [22]	ECC, hash, XOR	Password, smart card, and biometric-based authentication for IoT/MIoT	High computational cost
2014	Turkanovic et al. [23]	Hash, XOR	lightweight hash and XOR based authentication protocols for IoT	No untraceability; vulnerable to stolen smart card attacks
2024	Benfilali et al. [24]	Hash, XOR	User authentication for secure wireless sensor network access in IoT	Vulnerable to physical capture attacks
2021	Hussein et al. [25]	Hash, XOR	Lightweight secure remote user authentication for IoT applications	Vulnerable to physical capture attacks
2024	Safkhani et al. [26]	PUF, hash, XOR	Eavesdropping-resistant lightweight authentication for IoT environments	High computational cost
2025	Lee et al. [8]	PUF, hash, XOR	Lightweight authentication for MIoT environments	No untraceability; vulnerable to eavesdropping, stolen verifier, and ESL attacks
**2026**	**Proposed**	**PUF, hash, XOR**	**Secure and Lightweight Authentication Protocol for Medical IoT Environments**	**–**

**Table 2:** Notation.

Notation	Description
$U_{i}$	User
$SN_{j}$	Sensor node
GWN	Medical gateway node
$ID_{i}$	Identity of user
$PW_{i}$	Password
$PID_{i}$	Pseudo identity of the user
$SID_{j}$	Identity of the sensor node
$PSID_{j}$	Pseudo identity of the sensor node
$K_{gwn}$	Master key of the medical gateway
$K_{gu}$	Preshared key between user and gateway
$a_{i},b_{i},key_{i},x_{i},y_{i},z_{i},r_{1},r_{2},r_{3}$	Random nonces
$T_{i}$	Timestamps
SK	Session key
h(·)	Hash function
⊕	Exclusive OR operation
‖	Concatenation operation

**Table 3:** Notation of BAN logic.

Notation	Description
$N_{i},N_{ii}$	Principals
$S_{i},S_{ii}$	Statements
$N_{i}|\equiv S_{i}$	$N_{i}$ believes $S_{i}$
$N_{i}|∼S_{i}$	$N_{i}$ once said $S_{i}$
$N_{i}\Rightarrow S_{i}$	$N_{i}$ controls $S_{i}$
$N_{i}◃S_{i}$	$N_{i}$ receives $S_{i}$
$\#S_{i}$	$S_{i}$ is fresh
${\left\{S_{i}\right\}}_{K}$	$S_{i}$ is encrypted with *K*
$N_{i}\overset{K}{↔}N_{ii}$	$N_{i}$ and $N_{ii}$ have pre-shared key *K*

**Table 4:** Description of claim events in Scyther tool.

Claim Event	Description
Secrecy	Ensures the confidentiality of parameters exchanged during communication.
Aliveness	Verifies that the counterpart is actually participating in the communication.
Weak-agreement	Verifies that the counterpart is actually participating in the communication and is also a legal user.
Non-injective agreement (Niagree)	Verifies that the counterpart is actually participating in the communication, is a legitimate user, and agrees to the information exchanged.
Non-injective synchronization (Nisynch)	Verifies that the counterpart is actually participating in the communication, is a legitimate user, agrees to the information exchanged, and that the communication is performed correctly according to the protocol flow.

**Table 5:** Computational cost comparison of various protocols.

Protocol	User	Gateway	Sensor Node	Total	Cost (ms)
Subramani et al. [16]	$2T_{PUF}+2T_{Gen}+6T_{H}$	$2T_{Rep}+5T_{H}$	$2T_{PUF}+2T_{Gen}+6T_{H}$	$4T_{PUF}+4T_{Gen}+$ $2T_{Rep}+17T_{H}$	0.449
Shao et al. [17]	$T_{Rep}+T_{PUF}+11T_{H}$	$T_{Gen}+11T_{H}$	$T_{Rep}+2T_{PUF}+8T_{H}$	$3T_{PUF}+T_{Gen}$ $+2T_{Rep}+30T_{H}$	0.314
Hu et al. [21]	$2T_{ECM}+6T_{H}$	$1T_{ECM}+10T_{H}$	$2T_{ECM}+7T_{H}$	$5T_{ECM}+23T_{H}$	8.949
Huang [22]	$T_{Rep}+4T_{ECM}+17T_{H}$	$2T_{ECM}+17T_{H}$	$3T_{ECM}+8T_{H}$	$T_{Rep}+9T_{ECM}+42T_{H}$	15.817
Lee et al. [8]	$9T_{H}$	$7T_{H}$	$1T_{PUF}+4T_{H}$	$1T_{PUF}+20T_{H}$	0.087
Proposed	$11T_{H}$	$15T_{H}$	$2T_{PUF}+7T_{H}$	$2T_{PUF}+33T_{H}$	0.145

**Table 6:** Comparison of communication costs and messages.

Protocol	Communication Cost (bits)	Number of Messages
[16]	3520	5
[17]	4800	7
[21]	4608	4
[22]	5568	4
[8]	2816	4
Proposed	3712	4

**Table 7:** Energy consumption comparison of various protocols.

Protocol	$E_{C}$ (mJ)	$E_{M}$ (mJ)	$E_{\mathrm{total}}$ (mJ)
Subramani et al. [16]	4.885	2.323	7.208
Shao et al. [17]	3.416	3.168	6.584
Hu et al. [21]	97.365	3.041	100.406
Huang [22]	172.089	3.675	175.764
Lee et al. [8]	0.947	1.859	2.805
Proposed	1.578	2.450	4.028

**Table 8:** Comparison of security properties.

Security Property	[16]	[17]	[21]	[22]	[8]	Proposed
Eavesdropping Attack	∘	∘	∘	∘	×	∘
Untraceability	∘	∘	∘	∘	×	∘
Stolen Verifier Attack	∘	×	∘	∘	×	∘
Ephemeral Secret leakage Attack	∘	∘	×	∘	×	∘
User Device Capture Attack	∘	∘	∘	∘	∘	∘
Offline Password Guessing Attack	×	∘	∘	∘	∘	∘
Desynchronization Attack	∘	×	×	×	∘	∘
Impersonation Attack	×	×	×	∘	∘	∘
Perfect Forward Secrecy	∘	∘	∘	∘	∘	∘
PUF Modeling Attack	–	∘	–	–	∘	∘
Replay Attack	∘	∘	∘	∘	∘	∘
Man-in-the-Middle Attack	×	∘	×	∘	∘	∘
Denial of Service Attack	∘	∘	∘	∘	∘	∘
Mutual Authentication	∘	∘	∘	∘	∘	∘
Privileged Insider Attack	×	∘	×	∘	×	∘

*Note:* ∘indicates resistance to the corresponding attack or support for the corresponding security property, × indicates vulnerability or lack of the corresponding security property, and – indicates not applicable.

## Data Availability

The original contributions presented in this study are included in the article. Further inquiries can be directed to the corresponding authors.

## Abbreviations

The following abbreviations are used in this manuscript:

IoT	Internet of Things
MIoT	Medical Internet of Things
PUF	Physical Unclonable Function
XOR	Exclusive-OR
ESL	Ephemeral Secret Leakage
ROR	Real-or-Random
BAN	Burrows–Abadi–Needham
WSN	Wireless Sensor Networks
MitM	Man-in-the-Middle
ECC	Elliptic Curve Cryptography
CRPs	Challenge-Response Pairs
DY	Dolev–Yao
CK	Canetti–Krawczyk
DoS	Denial-of-Service
MMR	Meaning Rule
NVR	Nonce Verification Rule
JR	Jurisdiction Rule
BR	Belief Rule
FR	Freshness Rule
SPDL	Security Protocol Description Language
